# The Effects of Wastewater Treatment Plant Failure on the Gulf of Gdansk (Southern Baltic Sea)

**DOI:** 10.3390/ijerph19042048

**Published:** 2022-02-11

**Authors:** Marta Jaskulak, Maksymilian Sotomski, Małgorzata Michalska, Roman Marks, Katarzyna Zorena

**Affiliations:** 1 Department of Immunobiology and Environment Microbiology, Faculty of Health Sciences with Institute of Maritime and Tropical Medicine, Medical University of Gdansk, 80-210 Gdansk, Poland; maksymilian.sotomski@gumed.edu.pl (M.S.); malgorzata.michalska@gumed.edu.pl (M.M.); katarzyna.zorena@gumed.edu.pl (K.Z.); 2 Institute of Marine and Environmental Sciences, University of Szczecin, Mickiewicza 16, 70-383 Szczecin, Poland; roman.marks@usz.edu.pl

**Keywords:** wastewater, Gulf of Gdansk, wastewater treatment plant, wastewater release, raw wastewater, emergency discharge of sewage, Baltic Sea, total organic carbon, water pollution

## Abstract

In August 2019 and during August/September 2020, the main collection system of the Wastewater Treatment Plant (WWTP) in Warsaw, Poland, malfunctioned. During that system failure, over 4.8 million m^3^ of untreated wastewater was dropped directly into the Vistula River in just a few days. It is currently considered as one of the largest known failures of WWTP worldwide. In order to assess the environmental impact, water samples were collected from 2 spots at the Vistula river estuary (406 and 415 km from the discharge location, respectively), and 4 spots at the Gulf of Gdansk, situated on the southern shore of the Baltic Sea. The sampling was conducted before the wastewater wave reached the Vistula river’s mouth, followed by daily sampling during 21 days after the malfunction occurred. The study showed the decline in water quality at the Vistula river estuary and the Baltic shore waters as the wave of wastewater reached those points, despite being situated over 400 km downstream from the place of the accident. Those changes included the reduction in the dissolved oxygen content (by 0.69-fold at its peak), the increase in Total Organic Carbon (TOC) (by 1.28-fold at its peak), nitrate-nitrogen (N-NO_3_) (by 1.68-fold at its peak), phosphorous (P) (by 2.41-fold at its peak), conductivity (by 16.8-fold at its peak), and Chemical Oxygen Demand (COD) (by 1.84-fold). In the samples from the Vistula river, the decline in water quality was seen as incidental and lasted 2–3 days. Subsequently, the levels of physical and chemical parameters returned to the levels from before the accident. However, the changes in the Gulf of Gdańsk lasted significantly longer, especially on the West side of the Vistula river, where, even after 21 days from the initial accident, some parameters remained altered.

## 1. Introduction

“Czajka” Wastewater Treatment Plant (WWTP) is the main wastewater treatment facility in the capital city of Poland, Warsaw [1]. It is the largest and most modern wastewater treatment facility in Poland and in Eastern Europe, collecting raw wastewater from both sides of the capital city, which is divided by the Vistula River—the largest river in Poland. Czajka WWTP is situated on 56 ha of land and was initially completed in 1991, after almost two decades of construction [2]. However, when finally finished, the facility was already outdated and unable to deal with the rapidly increasing population of the capital city [3]. Extensive reconstruction, enlargement, and modernization were needed to meet the European Union (EU) standards for wastewater being implemented in 2007. It included the need to provide wastewater treatment for all urban effluents and to reduce the amounts of nitrogen (N) and phosphorus (P) in the wastewater released in the environment to a maximum of 10 mg/L and 1 mg/L, respectively [4]. Therefore, the facility was modernized and expanded in three stages, completed in the spring of 2013 [5]. Designed to support approximately 2.1 million people, the above-mentioned modernization doubled the facility’s daily inflow capacity from 200,000 m^3^ to 435,000 m^3^ per day and even to 515,000 m^3^ at peak times and emergencies [6]. Sewage sludge production also doubled to around 160,000 tons per annum [7]. The modernization cost, around 769 million euros (approximately USD 1 bn), was funded partially by the EU and by the Municipality of Warsaw [8].

On 28 August 2019, the transmission system between both sides of the Vistula river malfunctioned. A temporary replacement was constructed within the following 12 days, but approximately 3,600,000 cubic meters of untreated wastewater had been discharged directly into the Vistula river, making it one of the most significant system failures of any WWTP in the world. In 2019, before the first accident, 0.6 tons of P and around 10.8 tons of N were added to the Vistula river weekly by WWTP. During the accident, it is estimated that the Vistula river received around 10.8 t of P and 101 t of N per week [1]. A year later, another malfunction occurred, during which in a span of just a few days, over 4.8 million cubic meters of untreated wastewater was released directly into the Vistula River. At the begging of the malfunction, 3000 L of raw wastewater were released to the Vistula river every second. Due to heavy rainfall in the following days, that number rapidly increased to approximately 15–20 thousand cubic meters per second [4].

Given the sheer volume of raw wastewater released to the river, both failures of this WWTP were one of the largest WWTP accidents in the world. To date, the other significant malfunctions of WWTPs were the 2020 accident in Fort Lauderdale, Florida, U.S.A., with the release of over 1,045,000 m^3^ of raw sewage [9]; an accident in 2017 in Tijuana, Mexico, with a release of over 540,000 m^3^ wastewater into the Tijuana River; in 2006 in Waikiki, Hawaii, U.S.A., with the release of over 180,000 m^3^ of raw wastewater; an undetermined volume of raw wastewater released into the Casco Bay Portland, U.S.A. after an electricity failure and the malfunction of backup generators; and approximately a 140,000 m^3^ release of raw wastewater in 2020 during a WWTP failure in Seattle, Washington, U.S.A. [10,11]. In 2018, in Poland, there were emergency discharges of raw sewage into the Motława River, which then went into the Gulf of Gdańsk, Poland. As a result, 2300 m^3^ of untreated municipal wastewater was discharged into the Gulf of Gdańsk per hour. Our team attempted to assess the air and sea water after an emergency discharge of raw sewage into the Bay of Gdańsk. We presented the results of the research in several manuscripts [12,13,14,15].

The consequences of this event for the Vistula River and the Gulf of Gdańsk (situated on the southern side of the Baltic Sea) remain unknown. During both malfunctions, the Environmental Protection Inspection in Mazovia Voivodeship monitored the effects of the incident at several points from above the sewage drop until only 30 km down the river, which included the measurements of water temperature, dissolved oxygen content, water pH, conductivity, the total suspension, the content of nitrates and total nitrogen, chemical oxygen demand, and the total content of nitrogen, phosphorus, and carbon (https://wody.gov.pl/aktualnosci/1290-pilne-kolejna-awaria-w-oczyszczalni-sciekow-czajka-w-warszawie) (accessed on 12 January 2022). This study investigates the changes in surface water parameters before, during, and after WWTP malfunction at the Vistula river mouth (406–415 km downstream from the accident site) and in 4 points of the surface water on the Baltic seashore (in the subsequent few weeks).

Understanding and predicting the possible consequences of WWTP failure is fundamental now, since the majority of WWTP accidents occur due to a combination of structural failure as well as extreme weather conditions (such as heavy rains, floods, hurricanes, and earthquakes). It is estimated that approximately 72% of all WWTP accidents occur due to a combination of those reasons. Thus, due to the changing climate and extreme weather conditions occurring more commonly, it is estimated that the number of WWTP failures will continue to increase in the future [11].

The present study investigates the impact of pollutants, and the possible consequences of such malfunctions for the future. Since the source of contamination was municipal wastewater from a large city, the effects on eutrophication via an increase in the content of organic matter and nitrogen is of concern. In addition, the amount of dissolved oxygen (DO) is also measured as it is a crucial parameter for the organisms living in the water ecosystem. In addition, the Chemical Oxygen Demand (COD) is analyzed in the two sample points of the Vistula river to monitor the amount of organic matter. Last but not least, the changes in pH and water conductivity are also recorded.

In general, the Gulf of Gdańsk is an estuary of a considerable inflow of fluvial waters ranging from about 33.0 to 34.4 km^3^ per year. In addition, the Gulf of Gdańsk waters are permanently stratified [16,17], thus less dense riverine water tends to remain in the surface layer of estuary [18,19].

## 2. Materials and Methods

### 2.1. Sampling

**Figure 1:**
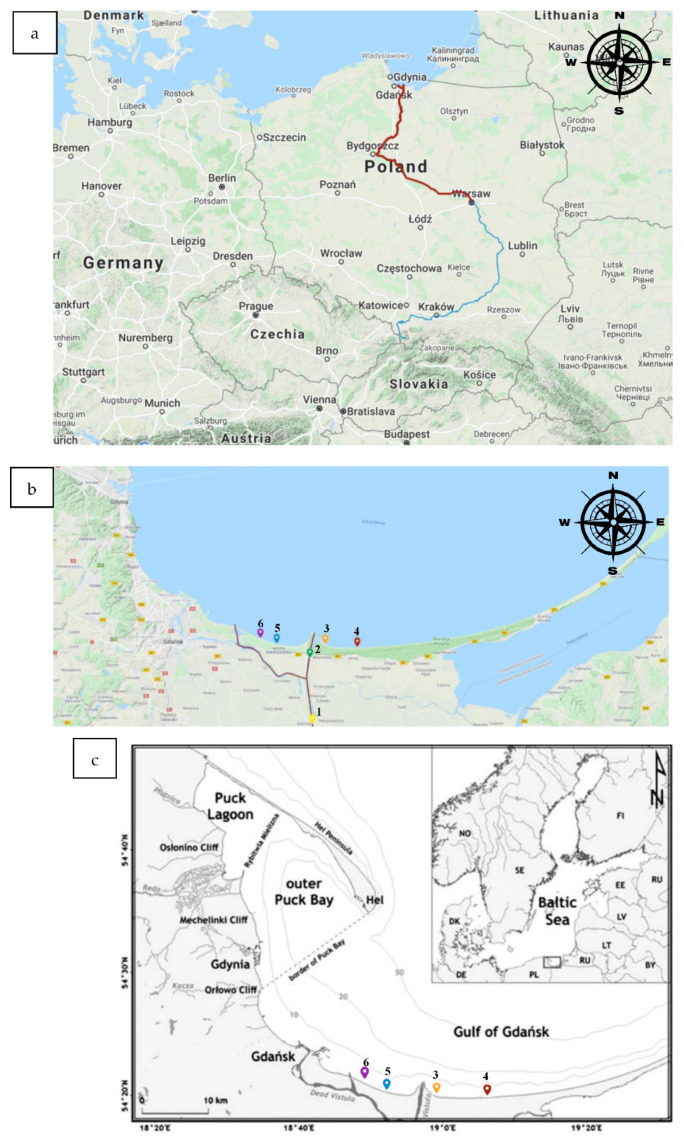
The map of Poland with the Vistula River. (**a**)—the Vistula River and the place of the accident. The point of the accident and the area downstream from the place of the accident is marked by a red line. (**b**)—sampling places at the end of the Vistula River and on the Gulf of Gdansk shore. (**c**)—Gulf of Gdańsk map with bathymetry. Surface water samples were collected at 6 points, i.e., (1) Kiezmark, (2) Mikoszewo-Wisła, (3) Mikoszewo Sea, (4) Jantar, (5) Wyspa Sobieszewska-Orle, and (6) Sobieszewo.

The sampling was performed in six sampling points (Figure 1). Two sampling points were situated at the estuary of the Vistula River (Point 1: GPS: 54.258176, 18.945701; Point 2: GPS: 54.333449, 18.939968; the distances from the site of the accident for Points 1 and 2 was: 406 and 415 km, respectively). The remining 4 sampling points were situated on the Southern shore of Baltic Sea in the Gulf of Gdansk region. Two of those points (Point 3 and 4) were situated on the eastern site of the Vistula River mouth (Point 3: GPS: 54.353164, 18.965191; Point 4: GPS: 54.344811, 19.032261; distance from the site of the accident: 421 and 426 km, respectively). The last two sampling spots were situated on the western side from the Vistula River mouth (Point 5 GPS: 54.352627, 18.935572 Point 6: GPS: 54.350606, 18.860966; the distances from the site of the accident: 421 and 423.5 km, respectively). All surface water samples were collected into 500 mL volume bottles previously sterilized and deionized. The sampling started on the 2 September 2020 and continued until the 9 September 2020, and the samples were collected daily. The last sample was collected on the 18 September 2020. Thus, the total number of days with sample collection was 9 (2.09.2020, 3.09.2020, 4.09.2020, 5.09.2020, 6.09.2020, 7.09.2020, 8.09.2020, 9.09.2020, and 18.09.2020). A total of 66 water samples were collected in the area of the mouth of the Vistula, to the Gulf of Gdansk. Surface water samples were collected at 6 points, i.e., Kiezmark (1), Mikoszewo-Wisła (2), Mikoszewo-Sea (3), Jantar (4), WyspaSobieszewska-Orle (5), and Sobieszewo (6) (Figure 1a). The water collection points are marked on the map (Figure 1b) and a map of the Gulf of Gdańsk with a bathymetry is presented in Figure 1c. All the samples were immediately stored in the fridge at temperature (+2 + 4 °C) and transported to the Medical University of Gdańsk, the Department of Immunobiology and Environmental Microbiology.

### 2.2. Meteorological Data

Meteorological data, including the water temperature, atmospheric pressure, wind speed, and direction, were obtained from the Institute of Meteorology and Water Management databases, National Research Institute, Poland (https://www.imgw.pl/) (accessed on 20 September 2020).

### 2.3. pH and Conductivity Assessment

The water pH was determined in accordance with the ISO 10523:2008 norm. The determination of the electrical conductivity was performed by following the ISO 7888:1985 norm. For both, the pH and the conductivity assessment, the Hanna instruments pH and conductivity meter Hi 9025 (HANNA INSTRUMENTS INC., Woonsocket, RI, USA) were used.

### 2.4. The Total Organic Carbon (TOC), Nitrate (N-NO_3_), and Total Phosphorus Content

The Total Organic Carbon (TOC) was measured by TOC Cell Test Merck Millipore kit (Merck & Co., Inc., Kenilworth, NJ, USA) by following the manufacturer’s instructions. In brief, sample digestion was performed with sulfuric acid and peroxydisulfate, which caused carbon-containing compounds to be transformed into carbon dioxide in the presence of the indicator. A total volume of 25 mL of each water sample was used for the assessment of TOC. The content of the total phosphorus was measured using Phosphate Cell Test, photometric, 0.5–25.0 mg/L (PO_4_^−^P), 1.5–76.7 mg/L (PO_4_^3−^), 1.1–57.3 mg/L (P_2_O_5_), Spectroquant^®^ (Merck & Co., Inc., Kenilworth, NJ, USA) by following the manufacturer’s instructions. The nitrate content was measured using the Nitrate Cell Test Method: photometric 0.5–18.0 mg/L NO_3_^−^N, 2.2–79.7 mg/L NO_3_^−^ Spectroquant^®^ (Merck & Co., Inc., Kenilworth, NJ, USA) by following the manufacturer’s instructions. The Dissolved Oxygen (DO) content was measured with oxygen Cell Test, photometric 0.5–12.0 mg/L O_2_ Spectroquant^®^ (Merck & Co., Inc., Kenilworth, NJ, USA) by following the manufacturer’s instructions. The Chemical Oxygen Demand for the samples located on the Vistula River was measured with the COD Cell Test photometric, 25–1500 mg/L (COD), Spectroquant^®^ (Merck & Co., Inc., Kenilworth, NJ, USA) following the manufacturer’s instructions.

## 3. Results

### 3.1. Meteorological Data

Table 1 presents the meteorological data from the sampling days at the mouth of the Vistula River. During the 2.5 weeks of sampling, the temperature oscillated between 17–19 °C. The atmospheric pressure was between 1013 and 1025 hPa, and the wind speed between 3–12 m/s. The wind direction varied for the first 4–5 days of sampling, and from 5 September the dominating direction of the wind was South-West.

### 3.2. Changes in the Physio-Chemical Parameters of the Water in the Vistula River after the Emergency Discharge of the Raw Sewage

**Figure 2:**
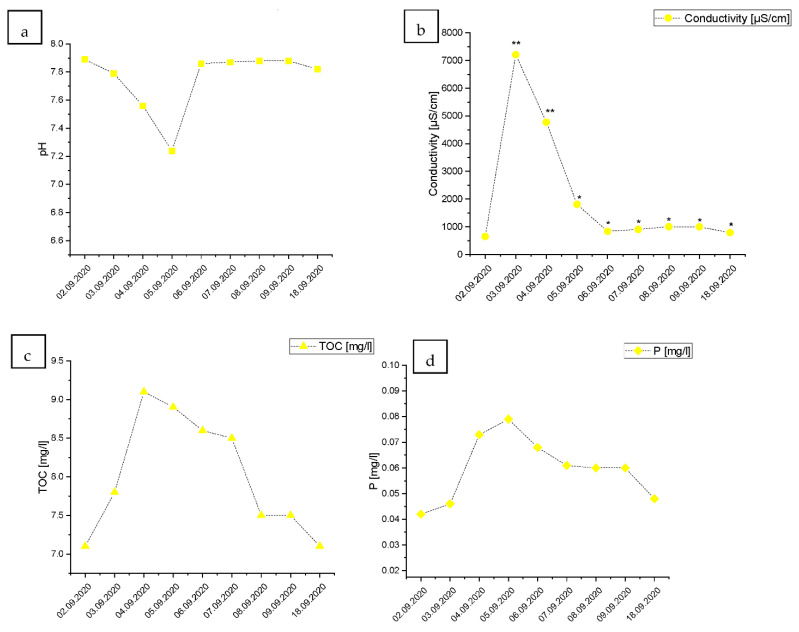

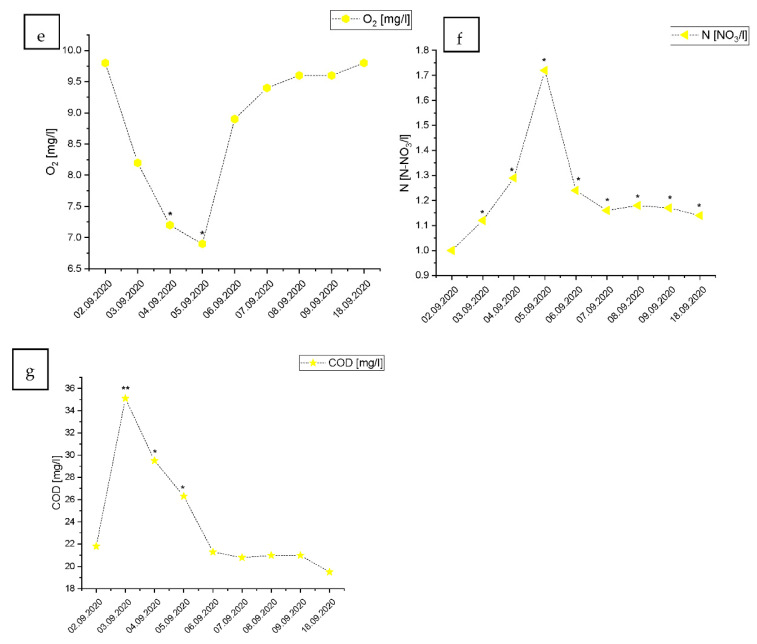
Changes in the water quality during the raw wastewater wave passage in Point 1 located at the Vistula River estuary (**a**): water pH, (**b**): conductivity [µs/cm], (**c**): TOC—total organic carbon [mg/L], (**d**): P—total phosphorus [mg/L], (**e**): dissolved oxygen content O_2_ [mg/L], (**f**): water N-NO_3_/L content, (**g**): COD—chemical oxygen demand [mg/L]). “*”—fall by one ecological class of water quality; “**”—fall by two ecological classes of water quality in comparison to the level measured at the beginning of the monitoring, based on the classification of the ecological status of surface water in accordance with the Ministry of Environment (2016), according to the classification of the status of the surface water bodies and environmental quality standards for priority substances (Polish Journal of Laws Dz. U. of 2016 item 1187).

Considering the points located on the Vistula River (Points 1 and 2), it is evident that the wave of the wastewater reached those points on the 3 September 2020 (Figure 2). At Point 1, the pH of the water continued to decrease from the initial 7.9 to 7.2 on 5 September. After that time, the pH returned to its initial state (Figure 2a). The water conductivity level increased over 11-fold in a span of 24 h between the 2 and 3 September, rising from 650 to 7210 µs/cm (Figure 2b). Such a dramatic increase indicates that during 2 and 3 of September, the water quality fell by two quality classes according to the classification of the ecological status of surface water in accordance with the Ministry of Environment (2016), according to the classification of the status of surface water bodies and environmental quality standards for priority substances (Polish Journal of Laws Dz. U. of 2016 item 1187) [20]. In the following days and until the end of sampling, water conductivity at Point 1 was still higher than on 2 September and belongs to class II of surface waters. The content of TOC rose from 7.1 to 9.1 mg/L at its peak during 4 September (Figure 2c). The elevated levels of TOC persisted similarly to the conductivity, until the end of sampling.

The content of total phosphorus reached its peak one day after the peak for TOC and two days after the peak of conductivity, on 5 September, and reached 0.079 mg/dm^3^, which was a 66.8% increase from the starting point (Figure 2d). The phosphorus levels remained elevated for a few consecutive days but decreased during the last measured time point. The raw wastewater release also decreased the content of dissolved oxygen. On 4 and 5 September, the dissolved oxygen levels decreased the water quality by 1 class point (Figure 2e). However, the dissolved oxygen levels increased after just four days and fully recovered by the final time point. The levels of nitrates increased during the raw sewage discharge from 1 mg to 1.72 on 5.09 (Figure 2f). After that, the levels of N-NO_3_ decreased but stabilized at an elevated level. The Chemical Oxygen Demand (COD) increased dramatically from 21.0 to 35.1 in just 24 h, causing a drop-in the water quality by two ecological classes (Figure 2g). However, the COD levels remained elevated for three days, after which the COD returned to the levels before the accident.

**Figure 3:**
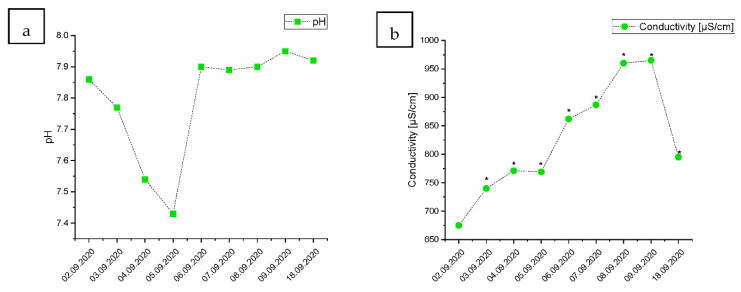

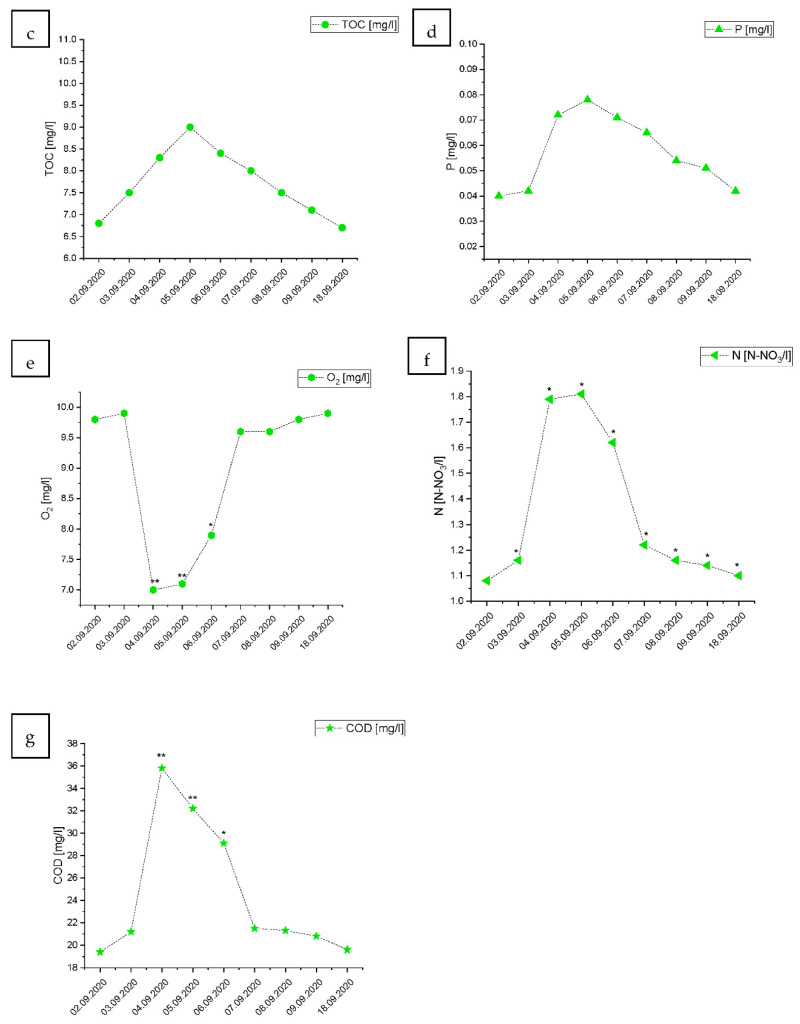
Changes in the water quality during the raw wastewater wave passage in Point 2 located at the Vistula River estuary (**a**): water pH, (**b**): conductivity [µs/cm], (**c**): TOC—total organic carbon [mg/L], (**d**): P—total phosphorus [mg/L], (**e**): dissolved oxygen content O_2_ [mg/L], (**f**): water N-NO_3_/L content, (**g**): COD—chemical oxygen demand [mg/L]). “*”—fall by one ecological class of water quality; “**”—fall by two ecological classes of water quality in comparison to the level measured at the beginning of the monitoring, based on the classification of the ecological status of the surface water in accordance with the Ministry of Environment (2016), according to the classification of the status of the surface water bodies and environmental quality standards for priority substances (Polish Journal of Laws Dz. U. of 2016 item 1187).

The results collected at Point 2, the last point on the Vistula River, before its release to the Gulf of Gdańsk on the southern Baltic Sea, were similar but delayed by approximately one day compared to Point 1. The pH levels decreased rapidly during the wave passage through, but bounced back up on 6 September (Figure 3a). The level of conductivity climbed for seven consecutive days, decreasing the water quality by one ecological class before starting to decrease on the last day of measurement (Figure 3b). The concentration measured on 18 September was still higher than on 2 September. Similarly, the concentration of TOC increased initially, but decreased by 18 September (Figure 3c). The phosphorus concentration increased by approximately 60–70%, compared to the conditions present before the wastewater release (Figure 3d). That increase lowered back to its original level on 18 September.

The Dissolved Oxygen level decreased rapidly, and for two consecutive days remained two ecological classes lower than before the wave (Figure 3e). However, the level of dissolved O_2_ bounced back relatively quickly and remained stable since 9 September. The level of N-NO_3_ increased by 59.67% at its peak and decreased again after just a few days (Figure 2f). The level of COD increased staggeringly from 19.4 to 35.8 at its peak, decreasing the water quality by II ecologic classification levels (Figure 2g). The level of COD remained elevated for three days, and afterwards, it declined to the level from before the sewage release.

### 3.3. Changes in the Physical and Chemical Parameters of Seawater on the Shore of the Gulf of Gdansk, East from the Vistula River Mouth

**Figure 4:**
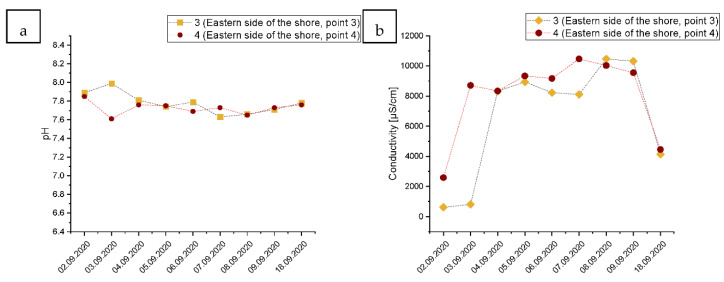

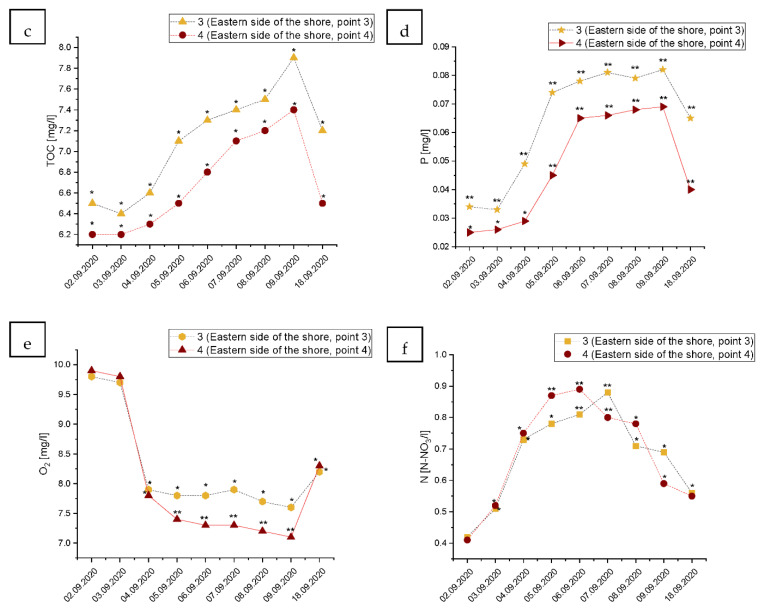
Changes in the water quality during the raw wastewater wave passage in Points 3 and 4 located on the eastern side from the Vistula River mouth in the Gulf of shore, where: (**a**): water pH, (**b**): conductivity [µs/cm], (**c**): TOC—total organic carbon [mg/L], (**d**): P—total phosphorus [mg/L], (**e**): dissolved oxygen content O_2_ [mg/L], (**f**): water N-NO_3_/L content. “*”—fall by one ecological class of water quality; “**”—fall by two ecological classes of water quality in comparison to the level measured at the beginning of the monitoring based on the classification of the ecological status of the surface water in accordance with the Ministry of Environment (2016), according to the classification of the status of the surface water bodies and environmental quality standards for priority substances (Polish Journal of Laws Dz. U. of 2016 item 1187).

The pH of the water from the western side of the estuary of the Vistula river, on the Gulf of Gdansk shore, lowered only slightly during the course of sampling without any significant changes (Figure 4a). However, the electrical conductivity of the water increased dramatically during the second day of the sampling and continued to increase during the following days. At its peak, the electrical conductivity in Point 3 increased over 14-fold after the wastewater entered the shore, compared to conditions before the accident. In Point 4, situated further west, a 3.8-fold increase in electrical conductivity was noticed (Figure 4b). For both points, the electrical conductivity lowered by 18 September, but did not reach the level from before the accident. In Point 3, the electrical conductivity on 18 September was still 5.33 times higher than before the accident and 1.8 times higher in Point 4.

The content of TOC on the western side of the Gulf of Gdansk Increased after the wave of wastewater from 6.5 mg C/dm^3^ to 7.9 mg C/dm^3^, and from 6.2 mg C/dm^3^ to 7.4 mg C/dm^3^ in Points 3 and 4, respectively (Figure 4c). During the last day of sampling, the total organic carbon content decreased in both points, but remained at a higher level compared to the point before the wastewater entered the shore.

The phosphorus content started to increase on 4 September and continued the trend continuously until 9 September. At its peak, the phosphorus content increased 2.5-fold and 2.8-fold in Points 3 and 4, respectively (Figure 4d). Similarly, to the record of TOC, the content of phosphorus was lower on the last sampling day but did not reach the state from before the accident.

The dissolved oxygen content decreased substantially from approximately 9.8, 9.9 mg/L on 2 September in Points 3 and 4, respectively, to approximately 7.6 and 7.8 mg/L on 4 September, decreasing the water quality by one ecological class (Figure 4e). In Point 3, the dissolved oxygen content remained stable until 9 September, while in point 4, the DO decreased further to approximately 7.1 mg/L on 9 September, resulting in the decrease in two ecological state levels. During the last sampling day, 18 September, the content of DO started to increase, but, similarly to the content of TOC and P, it did not reach the level from before the accident.

The nitrate-nitrogen content in the water on the eastern shore of the Baltic Sea more than doubled after the wave of wastewater, decreasing the overall water quality by two classes on 4, 5, and 6 September in Point 4, and on 6 and 7 September in Point 4 (Figure 4f). However, the nitrate-nitrogen content started to decline faster than the content of TOC, or P. The peak of the nitrate-nitrogen content was reached on 7 September in Point 3 and on 6 September in Point 4.

### 3.4. Changes in the Physical and Chemical Parameters of the Water on the Shore of the Gulf of Gdansk, West from the Vistula River Mouth

**Figure 5:**
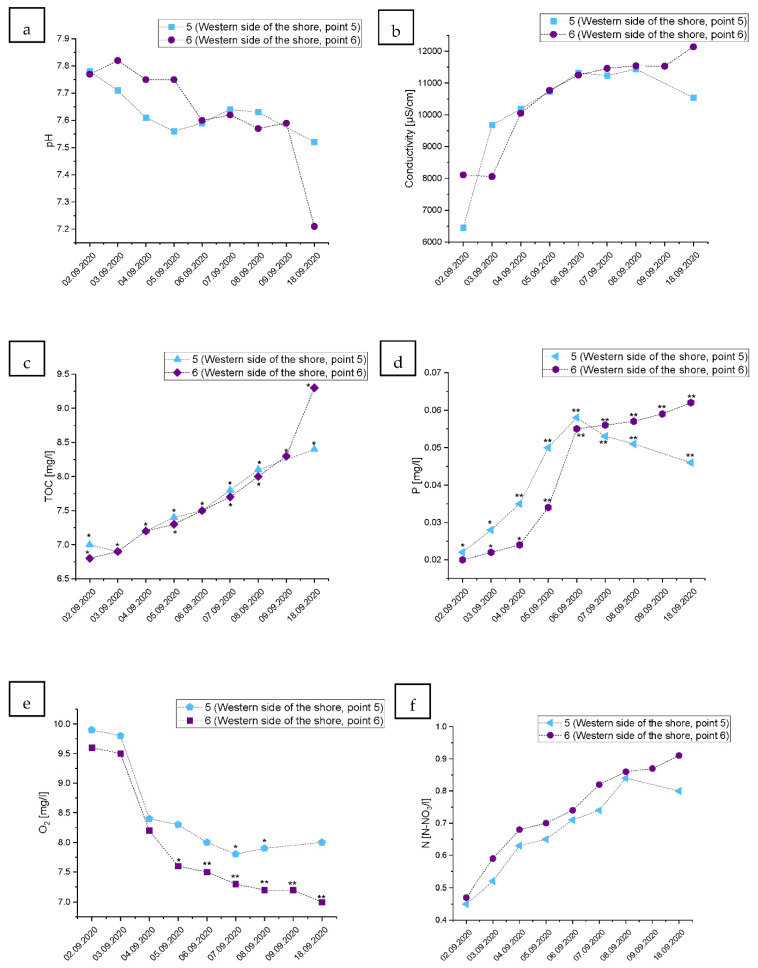
Changes in the water quality during the raw wastewater wave passage in Points 5 and 6 located on the western side from the Vistula River mouth in the Gulf of Gdansk shore, where: (**a**): water pH, (**b**): conductivity [µs/cm], (**c**): TOC—total organic carbon [mg/L], (**d**): P—total phosphorus [mg/L], (**e**): dissolved oxygen content O_2_ [mg/L], (**f**): water N-NO_3_/L content). “*”—fall by one ecological class of water quality; “**”—fall by two ecological classes of water quality in comparison to the level measured at the beginning of the monitoring based on the classification of the ecological status of the surface water in accordance with the Ministry of Environment (2016), according to the classification of the status of the surface water bodies and environmental quality standards for priority substances (Polish Journal of Laws Dz. U. of 2016 item 1187).

Interestingly, the physical and chemical consequences of the wastewater treatment plant failure were different on the west side from the Vistula river entry into the Gulf of Gdansk, in comparison to its east side. The pH of the water continued to decline through all sampling days, especially for Point 6 (further west) (Figure 5a). Similar effects were noticed for the changes in water conductivity (Figure 5b). The content of TOC continued to climb without any sign of decrease or return to the state from before the accident (in Points 5 and 6) (Figure 5c). The content of P started to decline at Point 5 but continued to increase through all the sampling timepoints in Point 6, causing a decrease in the water quality by two ecological classes (Figure 5d). Similarly, the N-NO_3_ content increased through all the sampling time points in Point 6 (Figure 5f). The dissolved oxygen content also continually decreased in Point 6, causing a drop-in the water quality by two ecological classes (Figure 5e). It is worth mentioning that Point 6 was located on the western side of the Gulf of Gdańsk, which is more isolated from the open Baltic Sea. In addition, during the sampling, the wind direction was west or southwest.

## 4. Discussion

The previous accidents of the Wastewater Treatment Plants showcased that in the event of the accident at the WWTP, the environment surrounding the facility, especially the waterbodies, are damaged [21]. The 2020 accident at the “Czajka” WWTP was the largest WWTP accident worldwide, in regard to the sheer volume of released wastewater [4]. Understanding its effects on the surrounding environment, the Vistula river, its estuary, and the Baltic Sea’s shore is crucial to understand the probable impact of such accidents in the future and designing ways to counteract its consequences. This is especially crucial now since over 72% of previous WWTP accidents were caused by a combination of structural failure with extreme weather conditions, which are estimated to occur more frequently in the future, leading to an increased chance of another major WWTP accident [11,22]. Emergencies caused by accidents in WWTPs are a significant and growing problem. The failure of any component of WWTP can lead to a decrease in the efficiency of wastewater treatment or even a failure of the entire facility, leading to the release of untreated wastewater into the environment. A crucial part of preventing WWTPs failure in the future and developing efficient ways of such emergency management is to uniform reporting of failures. Although most countries have an automatic system for failure reporting, the reports are not uniform and often do not include operational data [23]. Many components of WWTP can be subjected to operational failure, which can affect the primary process of wastewater collection, its transport, and treatment. However, most reports from the accidents are focused strictly on a technical point of view, starting from the failure instead of a system analysis point of view. Such a systematic analysis of the entire wastewater system performance and unified reporting could improve the performance of WWTP systems by addressing the weakest and critical components of the facility. In addition, a uniform registration of failure data has several more advantages. It also enables the cooperation of different authorities and makes the data exchange more accessible. A comparison between the performance of different facilities using the same systems can also be made, and any disturbances and the ways of their management can be easily identified. A failure data can then help to prevent the same failure in other facilities. Lastly, a uniform reporting system allows for the failure characteristics to be analyzed as soon as the data is available by operators and experts from other facilities, reducing the severity of the failure and allowing for faster response and management [24]. As an example, the most common cause of WWTP failure is reported as “device or structural failure”. However, such a cause includes a vast scope of possible subcategories, which are often not explained or unknown. Device or structural failure could include the failure of a pump, fan, or a loss of integrity of the pipe or the tank [25]. In another example, the collapse of the wastewater reservoir was classified as a structural failure, but the underlying cause, e.g., the aging of the materials or corrosion, was not reported, which is an essential issue for future safety management [26]. A crucial part of a risk assessment of any technology and operation is to perform a detailed analysis of the historical events associated with the specific operation and technology. Information on its weaknesses and previous failures in other places are extremely important. However, unfortunately, in most cases, such information is not available. An underestimated matter is that the wastewater itself is an effective degrader of some of the construction materials used due to the contents of chlorides, sulfates, and other substances [27]. Thus, during any reconstruction of a component of WWTP, special attention should be paid to the choice of the materials and the latest scientific knowledge about their resistance to certain chemicals. This can not only help to prolong the life of a WWTP, but also to prevent unexpected failures [28].

The most significant threat to the ecosystem and public health occurs in the closest proximity to the point of the accident [25]. Thus, the quality of water in the mixing zone below the discharge point on the Vistula river was controlled daily by the Regional Inspectorate for Environmental Protection. The results collected along the Vistula River showed exceed the parameters of DO and COD [4]. However, the accident’s impact on the river’s estuary and Gulf of Gdańsk was not researched in the past. The knowledge about the impact of such discharge can be used to assess possible risks associated with the accident to the environment and predict the possible consequences of similar accidents in the future [26]. The presented results showcase that the aquatic ecosystem in the Vistula river estuary and the nearshore of the Gulf of Gdansk is exposed to an excessive amount of discharged organic matter and nutrients after the emergency discharge of wastewater situated over 400 km upstream. In a similar study, the impact of raw wastewater discharge was assisted in the Danube river after a WWTP malfunction in Novi Sad, Serbia. The study results show severe water contamination approximately 7 km downstream from the accident point. However, the impact of the discharge on the water quality further downstream or at the river estuary was not researched. [29]. A study by Trávníček et al., in 2022, examined and compared recent WWTP failures and incidents worldwide. The study showed that the highest number of accidents affected the entire WWTP facility and were not isolated to a specific component. The most common manifestation of WWTP failure was the environmental damage that included the pollution of surface waters, groundwaters, soil, and even air when the biogas was released into the atmosphere during the accident [11].

Recent studies focused on the modeling predictions of wastewater fate, and their impacts showcased that the most significant threat to aquatic ecosystems is the discharge of inorganic nutrients, including nitrates, and phosphates. The second-largest threat to the aquatic ecosystems after wastewater discharge is reducing the dissolved oxygen content caused by the oxygen intake by organic matter. All of the changes mentioned above were identified as hazardous due to their accelerating effects on the eutrophication process, causing a further imbalance to the entire ecosystem [30]. However, in most cases, the long-distance sampling is not performed and the changes in those parameters on a distant stretch of rivers remain unknown. In the presented study, the content of nitrates and phosphorous was increased to over 400 km from the accident site, and the content of dissolved O_2_ was consequently decreased. The efficient removal of phosphorous and nitrogen from the municipal wastewater is of primary worldwide concern due to its impact on the eutrophication of water bodies that cause toxic algal blooms and hypoxia of the marine ecosystems, but also because of the risk to human health since the excessive concentrations of nitrates in the drinking water were linked to methemoglobinemia, thyroid diseases, diabetes, and gastric cancer [31,32]. This is an emerging concern since, in the past decades, the volume of produced wastewater and the need for more energy and food, as well as the application of fertilizers, have led to an increase in N and P in several waterbodies of great importance [33]. Nitrate has been a dominant form of increased N load since the 1970s and is usually removed by wastewater treatment facilities. In the present study, we noticed a spike in the nitrate content in the Vistula estuary after the failure of WWTP situated over 400 km upstream. However, that spike lasted only 2–3 days, after which the levels returned to normal, unlike the situation located even further from the accident site—along the eastern shore of the Gulf of Gdansk located east from the Vistula entry, where the levels of N-NO_3_ remained elevated for much longer before they started to decrease again. In contrast, on the western shore, the level of N-NO_3_ continued to climb for over 2.5 weeks without a sign of decreasing, indicating the presence of seawater being already mixed with wastewater. It is worth mentioning that excessive content of all forms of nitrogen has been shown to cause oxygen deficiency in water bodies, leading to deteriorating effects for aquatic organisms and increasing rates of eutrophication [9]. Moreover, the eutrophication problem of the southern Baltic Sea is primarily caused by anthropogenic activities that include the resale of untreated wastewater containing excessive amounts of nitrates, as well as from agricultural runoff into the sea [34,35]. Similarly, the P content is a crucial factor in eutrophication. In our study, its content was elevated by the release of raw wastewater in the Vistula River with the highest and most long-lasting effects situated on the western shore from the Vistula river mouth.

The increase in COD in the Vistula river after the WWTP was substantial, and decreased the quality of water at its peak by two classes, suggesting a sudden and large influx of organic pollutants despite the distance of the transport of the contaminated waters from the drop-point being over 400 km [36]. The influx of organic pollutants and excessive amounts of N and P caused a major drop in the DO content. Similar to the previous results, the situation on the Vistula river returned to its level before the accident rather quickly, whereas, on the western side of the Gulf of Gdansk seawater shore, it continued to deteriorate for over 2.5 weeks. However, even the short period of reduced O_2_ content can significantly affect the population of marine ecosystems, since the content of O_2_ is one of the most important abiotic factors in predicting the survival of fish in a given ecosystem, as well as being one of the most commonly used parameters for the assessment of water ecosystem health [37,38]. The collected experimental evidences indicate that especially organic pollutions after discharge to the seawater tend to surface and persist in the marine coastal environment for much longer. That points to the importance of the mixing conditions of wastewater with seawater, and the possible dispersion and mixing in the surrounding marine compartments.

The fate of pollution discharged into coastal seawater depends on the temporal and spatial wind fields that generate surface currents and wind waves. Both of them impact transport and mixing conditions at the surface, as well as within the whole water column. The inspection of the surface currents, obtained from the hydrodynamic model, revealed that during first two days (2 and 3 September) more easterly currents dominated, and were then replaced by more westerly directed transport along and towards the shore (from 4 to 12 September). Next, from 13 to 18 September, easterly currents again prevailed [39]. In addition, the suspended and dissolved organic compounds are scavenged by rising bubbles, especially in seawaters [40]. That process contributes to the formation of enriched microlayer [41,42]. Thus, the bubble mediated scavenge of organic compounds and its accumulation at the sea surface microlayer might be the reason why TOC data showed a more pronounced tendency to increase and slightly higher records along the western side of shore, compared with the values measured along the eastern side from the river mouth (Figure 4c and Figure 5c). Moreover, the presence of the surface microlayer suppresses the air-to-water-oxygen transfer, which resulted in enhanced oxygen depletion that also lasted longer at the western side (Figure 4e and Figure 5e).

## 5. Conclusions

The present study provides insights into the consequences of a major WWTP accident and sudden wastewater release into the river estuary’s ecosystem and the Baltic Sea’s shore. The presented results confirm that even over 400 km from the site of the accident, the receiving waterbody was highly affected and impaired by the release of raw wastewater and the discharged contaminants. Although the effects observed on the estuary of the Vistula River were resolved after a few days, the impacts resulted from the mixing of wastewater with the Baltic Sea, especially on the western side from the river’s entry into the Gulf of Gdansk, lasted much longer and showed no signs of improvement even 2.5 weeks after the wastewater release. This lead to the conclusion that in a case of a WWTP emergency, it is not only the ecosystem with the closest proximity that is highly affected. In such cases, the extent of the water testing should include the final recipient of a polluted waterbody. For example, the most affected point in our study was situated on the Baltic seashore, approx. 423.5 km from the site of the accident, 2.5 weeks after the accident. The study showed a 65.7% increase in the water P content, 56.3% increase in the content of N-NO_3_, 61.2% increase in water conductivity, and a drop in the content of dissolved oxygen by 37.1%. It was found that despite the distance of over 400 km from the place of the accident, the physical and chemical parameters of the river surface waters were significantly changed. However, more expanded impacts were established for the marine environments, where the dynamics of wastewater–seawater mixing and seawater circulation patterns impacted both the temporal and spatial changes.

## Figures and Tables

**Table 1:** Meteorological data from Gdańsk recorded by the Institute of Meteorology and Water Management, National Research Institute, Poland (https://www.imgw.pl/) (accessed on 20 September 2020).

Date (Day/Month)	Hour Time Zone: GMT + 1	Water Temperature °C	Atmospheric Pressure hPa	Wind Speed m/s	Wind Direction
1.09	6 AM	19	1018	5	N
2.09	6 AM	19	1014	9	NE
3.09	6 AM	19	1015	6	S
4.09	6 AM	18	1018	8	SW
5.09	6 AM	18	1013	6	SW
6.09	6 AM	18	1016	4	SW
7.09	6 AM	18	1019	3	SW
8.09	6 AM	18	1019	7	SW
9.09	6 AM	18	1018	4	SW
10.09	6 AM	17	1013	8	W
11.09	6 AM	17	1019	3	SW
12.09	6 AM	17	1016	3	S
13.09	6 AM	17	1023	7	W
14.09	6 AM	17	1025	5	SW
15.09	6 AM	17	1025	3	S
16.09	6 AM	17	1017	5	S
17.09	6 AM	18	1020	12	W
18.09	6 AM	18	1019	9	SW

## Data Availability

The raw data presented in this study are available on request from the corresponding author.

## References

[1] Piotrowska M., Dziewit L., Ostrowski R., Chmielowska C., Popowska M. (2020). Molecular Characterization and Comparative Genomics of IncQ-3 Plasmids Conferring Resistance to Various Antibiotics Isolated from a Wastewater Treatment Plant in Warsaw (Poland). Antibiotics.

[2] Oleszkiewicz J.A., Kalinowska E., Dold P., Barnard J.L., Bieniowski M., Erenc Z.F., Ones R.J., Rypina A., Udol J.S. (2004). Feasibility Studies and Pre-Design Simulation of Warsaw’s New Wastewater Treatment Plant. Environ. Technol..

[3] Wesołowska J. (2016). Urban Infrastructure Facilities as an Essential Public Investment for Sustainable Cities—Indispensable but Unwelcome Objects of Social Conflicts. Case Study of Warsaw, Poland. Transp. Res. Procedia.

[4] Preisner M. (2020). Surface Water Pollution by Untreated Municipal Wastewater Discharge Due to a Sewer Failure. Environ. Process..

[5] The National Programme for Municipal Waste Water Treatment. 2017—In Polish. https://www.kzgw.gov.pl/index.php/en/information-materials/programmes/the-national-programme-for-municipal-waste-water-treatment-npmwwt.

[6] (2018). Report on the Implementation of the National Municipal Wastewater Treatment Program in 2016 and 2017 (In Polish). https://bip.mos.gov.pl/strategie-plany-programy/krajowy-programzapobiegania-powstawaniu-odpadow/h/11170/051cde29/.

[7] Smol M. (2020). Inventory of Wastes Generated in Polish Sewage Sludge Incineration Plants and Their Possible Circular Management Directions. Resources.

[8] Kanownik W., Policht-Latawiec A., Wiśnios M. (2016). The Effect of Purified Sewage Discharge from a Sewage Treatment Plant on the Physicochemical State of Water in the Receiver. Annals of Warsaw University of Life Sciences—SGGW. Land Reclam..

[9] Griffin D.W., Banks K., Gregg K., Shedler S., Walker B.K. (2020). Antibiotic Resistance in Marine Microbial Communities Proximal to a Florida Sewage Outfall System. Antibiotics.

[10] Yuan S., Liu Z., Yin H., Dang Z., Wu P., Zhu N., Lin Z. (2019). Trace Determination of Sulfonamide Antibiotics and Their Acetylated Metabolites via SPE-LC-MS/MS in Wastewater and Insights from Their Occurrence in a Municipal Wastewater Treatment Plant. Sci. Total Environ..

[11] Trávníček P., Junga P., Kotek L., Vítěz T. (2022). Analysis of Accidents at Municipal Wastewater Treatment Plants in Europe. J. Loss Prev. Process. Ind..

[12] Michalska M., Zorena K., Bartoszewicz M. (2019). Analysis of Faecal Bacteria Isolated from Air and Seawater Samples Following an Emergency Sewage Discharge into the Gulf of Gdansk in 2018—Preliminary Study. Int. Marit. Health.

[13] Michalska M., Wąż P., Kurpas M., Marks R., Zorena K. (2021). Higher Number of Yeast-like Fungi in the Air in 2018 after an Emergency Discharge of Raw Sewage to the Gulf of Gdańsk—Use of Contingency Tables. Symmetry.

[14] Michalska M., Kurpas M., Zorena K., Wąż P., Marks R. (2021). Mold and Yeast-Like Fungi in the Seaside Air of the Gulf of Gdańsk (Southern Baltic) after an Emergency Disposal of Raw Sewage. JoF.

[15] Michalska M., Zorena K., Marks R., Wąż P. (2021). The Emergency Discharge of Sewage to the Bay of Gdańsk as a Source of Bacterial Enrichment in Coastal Air. Sci. Rep..

[16] Mikulski Z. (1970). Wody śródlądowe w strefie brzegowej południowego Bałtyku. (Inland waters in the Southern Baltic coastal zone). Prace Państw. Inst. Hydrol.-Meteorol..

[17] Majewski A. (1972). Charakterystyka hydrologiczna estuariowych wód u polskiego wybrzeża. (Hydrological characteristics of estuarine waters at the Polish Coast). Prace Państw. Inst. Hydrol.-Meteorol..

[18] Kowalewska-Kalkowska H., Marks R. (2015). Estuary, Estuarine Hydrodynamics. Encykl. Mar. Geosci..

[19] Lisimenka A., Kubicki A. (2019). Bedload transport in the Vistula River mouth derived from dune migration rates, southern Baltic Sea. Oceanologia.

[20] Ministry of Environment—Regulation of 21th July 2016 on the Classification of the Status of Surface Water Bodies and Environmental Quality Standards for Priority Substances. 2016 (In Polish). http://www.gios.gov.pl/images/dokumenty/pms/pms/SEM_Programme_2016-2020_ENG.pdf.

[21] Petroody S.S., Hashemi S.H., van Gestel C.A.M. (2020). Factors Affecting Microplastic Retention and Emission by a Wastewater Treatment Plant on the Southern Coast of Caspian Sea. Chemosphere.

[22] Naji A., Azadkhah S., Farahani H., Uddin S., Khan F.R. (2021). Microplastics in Wastewater Outlets of Bandar Abbas City (Iran): A Potential Point Source of Microplastics into the Persian Gulf. Chemosphere.

[23] Martínez R., Vela N., el Aatik A., Murray E., Roche P., Navarro J.M. (2020). On the Use of an IoT Integrated System for Water Quality Monitoring and Management in Wastewater Treatment Plants. Water.

[24] Hernández-Chover V., Castellet-Viciano L., Hernández-Sancho F. (2019). Cost analysis of the facilities deterioration in Wastewater Treatment Plants: A dynamic approach. Sustain. Cities Soc..

[25] Jaskulak M., Grobelak A., Vandenbulcke F. (2020). Modeling and optimizing the removal of cadmium by Sinapis alba L. from contaminated soil via Response Surface Methodology and Artificial Neural Networks during assisted phytoremediation with sewage sludge. Int. J. Phytoremediation.

[26] Herrera-Navarrete R., Colín-Cruz A., Arellano-Wences H.J. (2022). Municipal Wastewater Treatment Plants: Gap, Challenges, and Opportunities in Environmental Management. Environ. Manage..

[27] Makisha N. (2016). Restoration and Renovation of Waste Water Pumping Stations in Case of Emergency. Procedia Eng..

[28] Jaskulak M., Grobelak A. (2020). Soil enzymes in a changing climate. Climate Change and Soil Interactions.

[29] Lahijanzadeh A.R., Rouzbahani M.M., Sabzalipour S., Nabavi S.M.B. (2019). Ecological Risk of Potentially Toxic Elements (PTEs) in Sediments, Seawater, Wastewater, and Benthic Macroinvertebrates, Persian Gulf. Mar. Pollut. Bull..

[30] Perkins R., Whitehead M., Goulson D. (2021). Dead in the Water: Comment on “Development of an Aquatic Exposure Assessment Model for Imidacloprid in Sewage Treatment Plant Discharges Arising from Use of Veterinary Medicinal Products”. Environ. Sci. Eur..

[31] König M., Escher B.I., Neale P.A., Krauss M., Hilscherová K., Novák J., Teodorović I., Schulze T., Seidensticker S., Kamal Hashmi M.A. (2017). Impact of Untreated Wastewater on a Major European River Evaluated with a Combination of in Vitro Bioassays and Chemical Analysis. Environ. Pollut..

[32] Yamashita T., Yamamoto-Ikemoto R. (2014). Nitrogen and Phosphorus Removal from Wastewater Treatment Plant Effluent via Bacterial Sulfate Reduction in an Anoxic Bioreactor Packed with Wood and Iron. IJERPH.

[33] Zhang X., Zhang Y., Shi P., Bi Z., Shan Z., Ren L. (2021). The Deep Challenge of Nitrate Pollution in River Water of China. Sci. Total Environ..

[34] Xue Y., Song J., Zhang Y., Kong F., Wen M., Zhang G. (2016). Nitrate Pollution and Preliminary Source Identification of Surface Water in a Semi-Arid River Basin, Using Isotopic and Hydrochemical Approaches. Water.

[35] Jaskulak M., Rorat A., Kurianska-Piatek L., Hofman S., Bigaj J., Vandenbulcke F., Plytycz B. (2021). Species-Specific Cd-Detoxification Mechanisms in Lumbricid Earthworms Eisenia Andrei, Eisenia Fetida and Their Hybrids. Ecotoxicol. Environ. Saf..

[36] Hembach N., Alexander J., Hiller C., Wieland A., Schwartz T. (2019). Dissemination Prevention of Antibiotic Resistant and Facultative Pathogenic Bacteria by Ultrafiltration and Ozone Treatment at an Urban Wastewater Treatment Plant. Sci. Rep..

[37] Bossier S., Palacz A.P., Nielsen J.R., Christensen A., Hoff A., Maar M., Gislason H., Bastardie F., Gorton R., Fulton E.A. (2018). The Baltic Sea Atlantis: An Integrated End-to-End Modelling Framework Evaluating Ecosystem-Wide Effects of Human-Induced Pressures. PLoS ONE.

[38] Jaskulak M., Grobelak A., Vandenbulcke F. (2020). Effects of Sewage Sludge Supplementation on Heavy Metal Accumulation and the Expression of ABC Transporters in *Sinapis Alba* L. during Assisted Phytoremediation of Contaminated Sites. Ecotoxicol. Environ. Saf..

[39] (2019). Gulf of Gdańsk Currents. http://model.ocean.univ.gda.pl/php/frame.php?area=Baltyk.

[40] Marks R., Kruczalak K., Jankowska K., Michalska M. (2001). Bacteria and Fungi in Air over the Gulf of Gdańsk and Baltic Sea. J. Aerosol. Sci..

[41] Wurl O., Miller L., Röttgers R., Vagle S. (2009). The distribution and fate of surface-active substances in the sea-surface microlayer and water column. Mar. Chem..

[42] Engel A., Bange H.W., Cunliffe M., Burrows S.M., Friedrichs G., Galgani L., Herrmann H., Hertkorn N., Johnson M., Liss P.S. (2017). The Ocean’s Vital Skin: Toward an Integrated Understanding of the Sea Surface Microlayer. Front. Mar. Sci..

